# The Herald of Health

**Published:** 1866-07

**Authors:** 


THE HERALD OF HEALTH for July begins a new volume. Besides its usual variety of matter on physical culture and the cure of disease, this number contains original articles from Horace Greeley, Theodore Tilton, Rev. O. B. Frothingham, W. H. Burleigh, Dr. J. G. Webster, F. Beecher Perkins, G. W. Bungay and others. It also contains an original letter from Jeremiah Day, Ex-President of Yale College, now 94 years old, which should be read by all who would know how, by good habits, this man has lived so long. This magazine begins its new volume with 16 additional pages. Its motto is: A higher type of manhood, physically, morally and intellectually. $2 a year, 20 cts. a number. The seven numbers of this year, now ready, sent as samples for 70 cents. Address Miller, Wood & Co., 15 Laight street, New York.

